# Patent Ductus Arteriosus Associated with Congenital Anomaly of Coronary Artery

**DOI:** 10.5812/cardiovascmed.12281

**Published:** 2013-10-28

**Authors:** Majid Maleki, Nassrin Azizian, Maryam Esmaeilzadeh, Bahieh Moradi

**Affiliations:** 1 Echocardiography Research Center, Rajaie Cardiovascular Medical and Research Center, Iran University of Medical Sciences, Tehran, IR Iran

**Keywords:** Patent Ductus Arteriosus, Congenital Abnormalities, Coronary Vessels, Coronary Angiography

## Abstract

We reported a case of patent ductus arteriosus (PDA) with congenital anomaly of coronary arteries as abnormal origin of right coronary artery (RCA) and left coronary artery (LCA) from a single ostium of the right coronary sinus. A 21-year-old man referred to our institution for evaluation of cardiac murmur. He has suffered from palpitation and atypical chest pain for three months. On physical examination, a continuous murmur was heard in the second left parasternal space. Transthoracic echocardiography showed normal left and right ventricular size and systolic function (LVEF = 55%). Main pulmonary artery (PA) and left pulmonary artery (LPA) branch were considerably dilated. Considering normal coronary flow, lack of clinical evidence of myocardial ischemia and echocardiography findings, patient underwent surgical closure of PDA via left thoracotomy and after five days discharged uneventfully.

## 1. Introduction

We reported a case of patent ductus arteriosus (PDA) with congenital anomaly of coronary arteries as abnormal origin of right coronary artery (RCA) and left coronary artery (LCA) from a single ostium of the right coronary sinus. 

## 2. Case report

**Figure 1.**
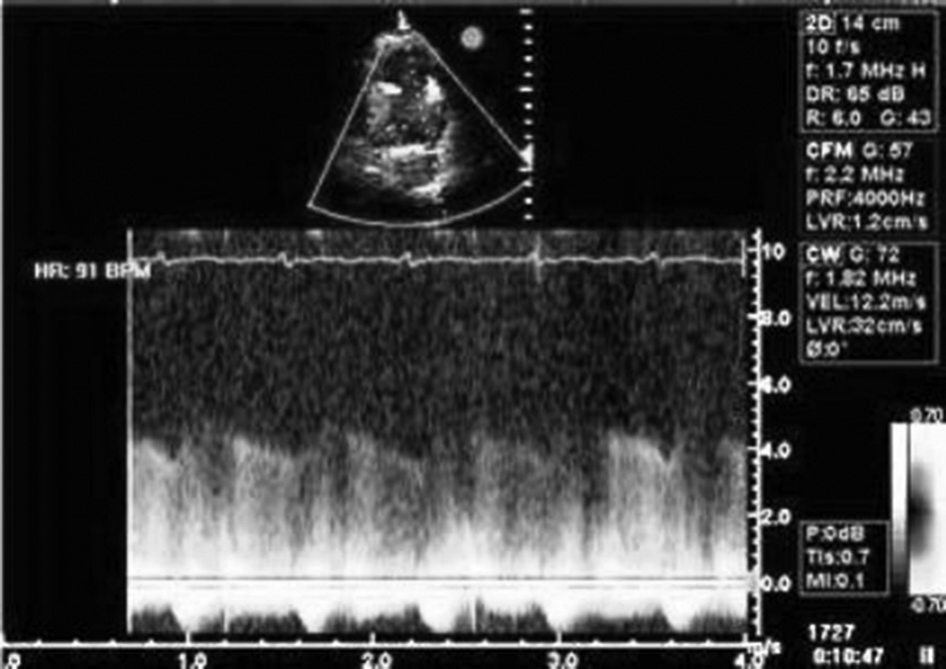
Parasternal short axis view shows high velocity continuous flow of patent ductus arteriosus

**Figure 2.**
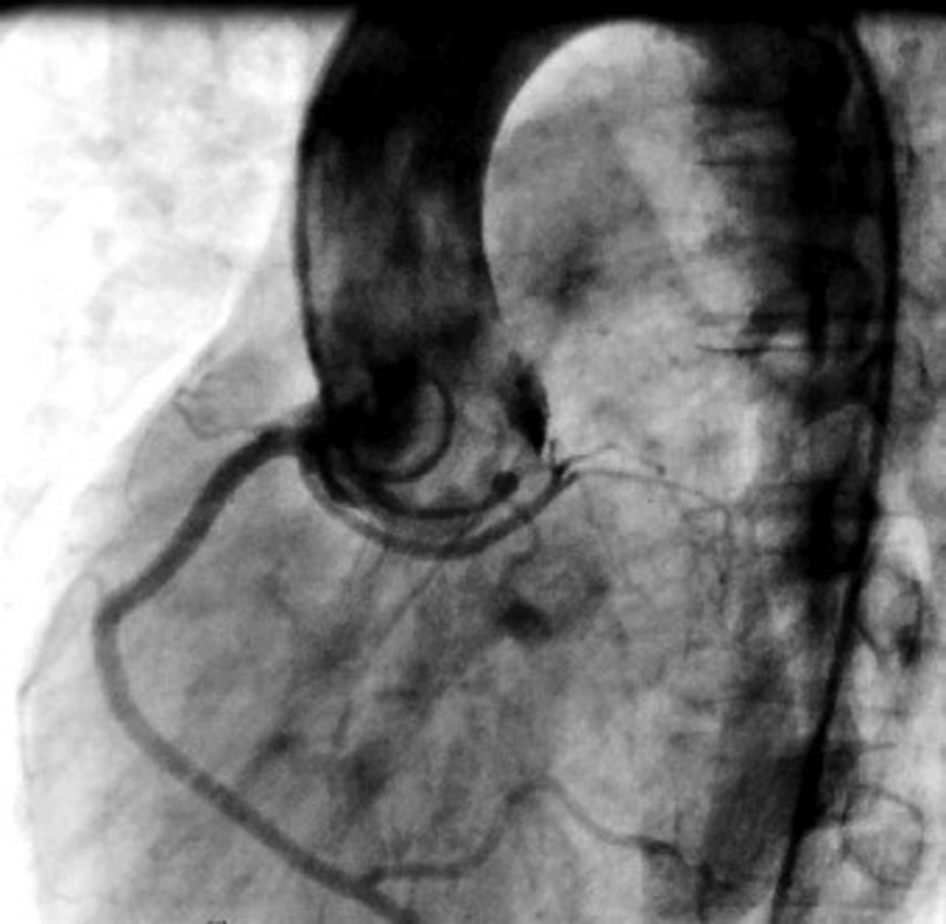
Aortography in left anterior oblique view shows abnormal origin of LCA (left coronary artery) and RCA (right coronary artery) from a single ostium of right sinus of valsalva

A 21-year-old man referred to our institution for evaluation of cardiac murmur. He has suffered from palpitation and atypical chest pain for three months. On physical examination, a continuous murmur was heard in the second left parasternal space. Transthoracic echocardiography showed normal left and right ventricular size and systolic function (LVEF= 55%). Main pulmonary artery (PA) and left pulmonary artery (LPA) branch were considerably dilated. A small (5-6mm) PDA with insignificant left to right shunt was detected (Figure 1) without the evidence of increased pulmonary pressure (systolic PAP = 30mmHg). Patient was scheduled for device closure. Cardiac catheterization and O _2_ saturation study showed normal RV pressure and pulmonary artery pressure. Aortic root injection depicted that both RCA and LCA originate from the right coronary sinus through a single ostium, LAD was located superiorly with a benign anterior course (Figure 2). Aortography showed small PDA, but because of aneurysmal dilation of main PA and LPA, transcutaneous device closure was technically difficult and unsuccessful. Considering normal coronary flow, lack of clinical evidence of myocardial ischemia and echocardiography findings, patient underwent surgical closure of PDA via left thoracotomy and after five days discharged uneventfully. 

## 3. Discussion

**Figure 3.**
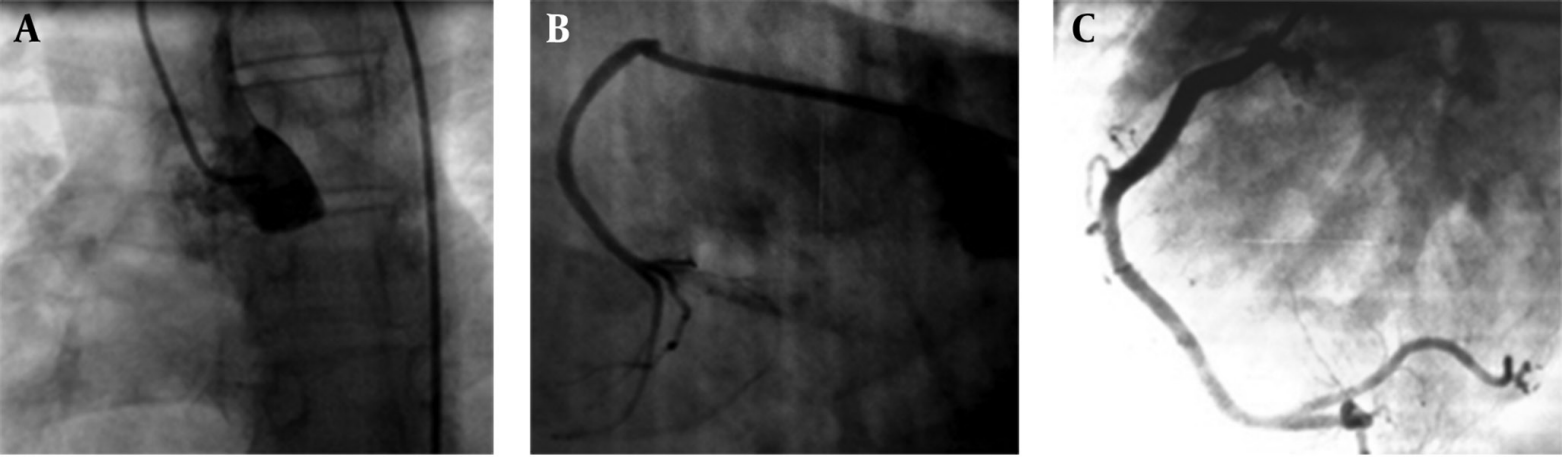
Selective coronary angiography in left anterior oblique views show absence of coronary ostium in left sinus of valsalva (A) and abnormal origin of LCA (B) and RCA (C) from a single ostium of right sinus of valsalva

In an anatomic collection of congenital heart disease by Frescura et al. , an isolated anomalous origin of coronary arteries was reported to be 2.2% (27 of 1,200 specimens), both right and left coronary artery from the right sinus were reported to be 15% (4 of the 27 patients) ( 1 ). In review of two large registries (in the U.S. and Italy) of young competitive athletes who died suddenly, Basso et al. reported 27 sudden deaths in young athletes, identified solely at autopsy due to either left main coronary artery from the right aortic sinus (n = 23) or right coronary artery from the left sinus (n = 4) (2). Patent ductus arteriosus (PDA), the most common type of extra cardiac shunt, represents persistent patency of the vessel that normally connects the pulmonary arterial system and the aorta in the fetus. The ductus arteriosus usually closes within two or three days of birth, but may remain patent for a lifetime. Diagnosis of the PDA can be made using echocardiography or cardiac catheterization. When catheterization is performed, the catheter usually passes quite easily through the pulmonary artery to the descending aorta. To determine the origin of the coronary arteries, Dotan et al. (3 ) reviewed 206 angiograms of PDA patients which were obtained between 1999 and 2011. In 49.5 % of patients with adequately visualized coronary arteries’ origin, an anomalous origin of coronary arteries was detected in 10.8 % of patients (11 of the 102 patients) , amongst them, single common coronary artery origin is considered as the most common abnormality (6.8 %). One patient had an aberrant origin of the left coronary artery from the noncoronary sinus, and three patients had an aberrant origin of the right coronary artery: two from the left coronary sinus and one from the noncoronary sinus. Their findings suggest that the incidence of coronary artery anomalies associated with isolated PDA may be substantially much higher than what was previously reported. Regarding the increased risk for sudden death with coronary anomalies, a reasonable approach is to determine the coronary artery origin and pathway after the diagnosis of an isolated PDA. Reports of PDA associated with the abnormal origin of the coronary arteries from the right coronary are even far rare. Recognition of the coronary anomaly before ligation of the duct is important and the consequence of routine ligation may be disastrous. The left heart catheterization to detect coronary arteries should be performed in PDA patients particularly before either intervention or surgery. When the origins of the coronary arteries are appeared abnormally, selective coronary angiography is mandatory (Figure 3).
